# SARS-CoV-2 in Mozambican primary school-aged children at Maputo City and Province: a cross-sectional study from a low-income country

**DOI:** 10.1186/s12887-024-04904-x

**Published:** 2024-07-02

**Authors:** Adilson Fernando Loforte Bauhofer, Édio Ussivane, Assucênio Chissaque, Fátima Iahaia, Ramígio Pololo, Fernanda Campos, Emerson Miranda, Luciana António, Plácida Maholela, Aline Gatambire, Marlene Djedje, Fátima Ráice, Luzia Gonçalves, Nilsa de Deus, Osvaldo Inlamea

**Affiliations:** 1 Instituto Nacional de Saúde (INS), EN1, Bairro da Vila-Parcela, Distrito de Marracuene, Província de Maputo, Mozambique; 2 Instituto de Higiene e Medicina Tropical, Universidade Nova de Lisboa, Rua da Junqueira, Lisboa, Portugal; 3 Centro de Estatística e Aplicações da Universidade de Lisboa, Lisboa, Portugal; 4 Departamento de Ciências Biológicas, Universidade Eduardo Mondlane, Maputo, Mozambique; 5 z-Stat4life, Lisboa, Portugal

**Keywords:** Prevalence, SARS-CoV-2, School-aged children, Maputo-Mozambique

## Abstract

**Background:**

Seroprevalence studies provide information on the true extent of infection and capture demographic and geographic differences, indicating the level of immunity against Severe Acute Respiratory Syndrome Coronavirus 2 (SARS-CoV-2). We sought to provide local evidence of SARS-CoV-2 exposure in school-aged children during in-class teaching in Maputo City and Province, Mozambique.

**Methods:**

Between August and November 2022, we performed a cross-sectional study in school-aged children in four schools in rural, peri-urban, and urban areas of Maputo City and Province. A point-of-care test was used to evaluate SARS-CoV-2 antigens and anti-SARS-CoV-2-specific immunoglobulin M (IgM) and immunoglobulin G (IgG) antibodies. Descriptive statistics were used to estimate the prevalence of the antigens and antibodies. Multiple logistic regression models were used to estimate the adjusted odds ratio (AOR) for the factors associated with anti-SARS-CoV-2 antibodies.

**Results:**

A total of 736 school-aged children were analyzed. The prevalence of the SARS-CoV-2 antigen was 0.5% (4/736). The prevalence of SARS-CoV-2 antigens was 0.0% (0/245), 0.8% (2/240) and 0.8% (2/251), in the rural, peri-urban and urban areas respectively. The overall seroprevalence of the anti-SARS-CoV-2 antibodies (IgG or IgM) was 80.7% (594/736). In rural area anti-SARS-CoV-2 IgG or IgM antibodies were detected in 76.7% (188/245), while in peri-urban area they were detected in 80.0% (192/240) and in urban area they were detected in 85.3% (214/251). In the adjusted logistic regression model, school-aged children from the urban area were more likely to have anti-SARS-CoV-2 IgG or IgM antibodies than were school-aged children from the rural area (adjusted odds ratio: 1.679; 95% CI: 1.060–2.684; p-value = 0.028).

**Conclusions:**

During the in-class teaching period, active SARS-CoV-2 cases in school-aged children were observed. More than half of the school-aged children were exposed to SARS-CoV-2, and SARS-CoV-2 was significantly more common in the schools at the urban area than in the school in the rural area at Maputo City and Province.

**Supplementary Information:**

The online version contains supplementary material available at 10.1186/s12887-024-04904-x.

## Introduction

The emergence of the Coronavirus disease (COVID-19) pandemic, caused by the Severe Acute Respiratory Syndrome Coronavirus 2 (SARS-CoV-2) [1], has led to the introduction of non-pharmacological interventions, such as the closure of schools with the aim of limiting virus transmission, slowing community transmission, and preventing health care system saturation and subsequent collapse [2, 3]. School closures have negatively affected students, with an estimated 100 million falling below the minimum proficiency level in reading [4].

Mozambique, a sub-Saharan African country, reported its first COVID-19 case in March 2020 [5] and, as implemented in other settings, implemented a complete closure of schools in 2020 [6], in which the prevalence of anti-SARS-CoV-2 antibodies in children attending primary schools ranged from 0.0 to 7.9% in the main capital cities of each province. The data were generated from a national community survey conducted in locations with high population density and among key groups (e.g., health workers) between July and August 2020 [7, 8].

Increased seroprevalence over time has been reported in different settings, primarily due to the emergence of variants of concern, which led to the reporting of different waves [9–11]. Mozambique reported four waves from June 2020 to January 2022 [12], and within that time, three cross-sectional studies in a rural area in the southern region of the country indicated that the seroprevalence increased from 25.6% (April-June 2021) to 58.2% (October-November 2021) and later to 82.6% (January-February 2022) in children and adolescents aged 0–19 years [13].

Our knowledge of the actual extent of SARS-CoV-2 infection in Mozambican children can be underestimated, as antibodies can decline over time [14, 15], increasing the number of susceptible groups, such as school-aged children. In addition, most cases of COVID-19 are symptomatic and standard monitoring systems often fail to capture the full extent of exposure, as they only confirm identified symptomatic cases in areas with adequate diagnostic resources. Further seroprevalence surveys could help define the trends and quantify the extent of the exposure among the at-risk population considering the emergence of different variants of concern and rate of antibodies decline, to better plan public health interventions [16], such as prioritizing vaccination for the most at-risk groups. Secondly, identifying active cases within schools can help identify potential viral transmitters, their characteristics, and the possibility of ongoing community transmission. This study aimed to provide an updated estimate of SARS-CoV-2 exposure among school-aged children from four primary schools in Maputo City and Province, Mozambique.

## Methods

### Settings

This study was conducted between August and November 2022 at primary schools in rural, peri-urban and urban areas in Maputo City and Province in Mozambique. The urban area in Maputo City has 61 primary schools with a total of 38,191 school-aged children, and the peri-urban area has 124 primary schools with a total of 146,225. The rural area in Marracuene district in Maputo Province has 48 primary schools with a total of 59,081 school-aged children.

Before the onset of this study and since the onset of COVID-19, Mozambique reported 229,564 COVID-19 cases and 2,215 deaths [17]. COVID-19 immunization in the country began in the first quarter of 2021 for people aged 15 years or older [18].

None of the enrolled schools had a surveillance or monitoring system that would enable tracking and registration of suspected/confirmed SARS-CoV-2 cases.

### Study design and population

A cross-sectional study was conducted in public primary schools in Maputo City and Province. The study population consisted of primary school-aged children from Maputo City and Province in Mozambique who were unvaccinated for COVID-19.

### Sample size

The binomSamSize package was used by applying Wilson’s method instead of the Wald method to determine the sample size for a binominal proportion [19, 20]. A 3.1% seroprevalence from a previous survey during lockdown [8], a margin error of 2.5% for a 95% confidence interval (CI), was considered, and the minimum sample size needed for each area (rural, peri-urban and urban) was 237 school-aged children, totaling a minimum of 711 children. After accounting for 5% nonresponse, the final sample size was 746.

### Sampling

A two-step approach was used to select the settings and study participants. In the first step, one school was selected from rural, peri-urban, and urban community. However, where the targeted sample size was not met in a purposively selected school, a nearby school was purposively selected and sampled until the target was met. Purposively sampling was used owing to logistics constraints, for example, available funding for transport of the study material for rapid diagnosis testing and disposability of the generated biological waste. The rural area school was the only one that had indoor and outdoor classes, limited water access, and no water cistern.

In the second step, all the legal guardians of the school-aged children in the selected schools were invited to attend the study presentation, after the presentation legal guardians were asked to indicate their availability for the study procedures. Legal guardians who were not present in the study presentation were contacted by phone to explain the study procedures and availability to participate was recorded. At the date of the visit, primary school-aged children who were accompanied by their legal guardian were consecutively enrolled, upon arrival at school until the targeted sample size was achieved.

### Study procedures

After the confirmation of availability of the legal guardian and the respective child, a booking log was set. The day before the schedule visit day, a courtesy phone call was made to the legal guardian reminding them of the scheduled visit. On the day of enrollment, the study objectives and procedures were re-explained to the legal guardian and the child, and any queries from the legal guardian and the child were resolved. Written informed consent was obtained from each participant’s legal guardian, and informed assent was obtained from every participant older than 11 years.

A semi structured form (Additional file 1) was used to collect the data by interviewing the school-aged children and their legal guardians. The recorded data included information on participants’ sex, age in years, number of household members, whether participants covered their mouth and nose with a bent elbow (in a “V” shape) when coughing or sneezing, whether participants slept in an environment with a social distance of 1.5 m from other people in the household, whether participants used public transport to attend school, whether participants had contact with a confirmed SARS-CoV-2 infection in their household or school, and whether participants had a positive SARS-CoV-2 diagnosis prior to enrollment in the study.

Health professionals, biologists, laboratory technicians and nutritionists were trained to collect and perform point-of-care rapid tests at school according to the manufacturer’s recommendations (Panbio, COVID-19, Abbott, Jena, Germany) [21, 22] to detect SARS-CoV-2 antigens through nasal swab collection and anti-SARS-CoV-2 specific immunoglobulin G (IgG) and immunoglobulin M (IgM) antibodies through capillary blood collection. The antigen test had 98.1% and 99.8% sensitivity and specificity, respectively [21], while the antibody test had 96.2% sensitivity and 100.0% specificity for fingerstick whole blood collection [22]. Participants detected to be positive for SARS-CoV-2 antigens were advised to follow the country’s guidelines, which recommended home isolation for seven days if asymptomatic or referral to the nearest hospital to seek health care if symptomatic. All collected data were recorded in the Open Data Kit application on a secure tablet or smartphone.

### Statistical analysis

Data analysis was conducted using R software, version 4.1.0 (Vienna, Austria) [23]. A listwise case deletion procedure was performed. Descriptive statistics were used to describe the population (Table 1). Four outcome variables were evaluated: anti-SARS-CoV-2 IgG or IgM; anti-SARS-CoV-2 IgG; anti-SARS-CoV-2 IgM; and SARS-CoV-2 antigen. Outcome variables are reported as proportions with Wilson’s 95% confidence intervals.

**Table 1:** Characteristics of school-aged children enrolled in Maputo City and Province between August and November 2022

Characteristic	*N* = 736		
**Area**			
Rural	33.3% (245)		
Peri-urban	32.6% (240)		
Urban	34.1% (251)		
**Sex**			
Male	47.6% (350)		
Female	52.4% (386)		
**Age in years, Median (Q1 - Q3) (Min - Max)**	8 (7 - 10) (5 - 15)		
**Number of household members, Median (Q1 - Q3) (Min - Max)**	5 (4 - 7) (2 - 15)		
**Does the child cover’s mouth and nose with a bent elbow when coughing/sneezing?**			
Yes	67.5% (497)		
No	32.5% (239)		
**Does the child sleeps 1.5 m away from other beds?**			
Yes	34.8% (256)		
No	65.2% (480)		
**Does the child use public transport to school?**			
Yes	23.8% (175)		
No	76.2% (561)		
**Did the child had contact with a SARS-CoV-2 case at home?**			
Yes	15.1% (111)		
No/Doesn’t know	84.9% (625)		
**Did the child had contact with a SARS-CoV-2 case at school?**			
Yes	6.4% (47)		
No/Doesn’t know	93.6% (689)		
**Did the child had a SARS-CoV-2 positive diagnosis before enrollment in the study?**			
Yes	0.7% (5)		
No	99.3% (731)		

Independent variables, including potential confounding variables or effect modifiers, were: participants’ sex, age in years, number of household members, whether participants covered their mouth and nose with a bent elbow when coughing or sneezing, whether participants slept in an environment with a social distance of 1.5 m from other people in the household, whether participants used public transportation to attend school, whether participants had contact with a confirmed SARS-CoV-2 infection in their household or school, and whether participants had previous infection by SARS-CoV-2.

Crosstabulation between the potential confounding variables or effect modifiers and the outcome variables were performed, except for the SARS-CoV-2 antigen outcome. Unadjusted and adjusted odds ratio values were estimated using logistic regression models. The *step()* function in R was used to generate the final multiple logistic regression model based on the model with the lowest Akaike information criteria (Table 2 and Additional files 2 and 3). P-values less than 5% were considered to indicate statistical significance.

## Results

### Participant characteristics

From August to November 2022, 746 participants were enrolled in this study. After the listwise case deletion procedure, the final sample size was 736, of whom 33.3% (245/736) were from rural area, 32.6% (240/736) were from peri-urban area and 34.1% (251/736) were from urban area. The median age in years was eight years, ranging from five to 15 years. Contact with a SARS-CoV-2 case at home was observed in 15.1% (111/736) of respondents, whereas contact with SARS-CoV-2 at school was observed in 6.4% (47/736, Table 1).

Of the participants who had a SARS-CoV-2 positive diagnosis before enrollment in this study (0.7%; 5/736), one was hospitalized, four had headaches, three had fever, two had fever and nasal congestion, and one had dizziness.

### Prevalence of SARS-CoV-2 antigens and anti-SARS-CoV-2 antibodies

During the study period SARS-CoV-2 antigens were detected in 0.5% of the enrolled school-aged children (4/736; 95% CI: 0.2–1.4). In rural, peri-urban, and urban areas, the prevalence of SARS-CoV-2 antigens was 0.0% (0/245; 95% CI: 0.0–1.5), 0.8% (2/240; 95% CI: 0.2–3.0) and 0.8% (2/251; 95% CI: 0.2–2.9), respectively.

The seroprevalence of anti-SARS-CoV-2 IgG or IgM antibodies was 80.7% (594/736; 95% CI: 77.7–83.4), that of anti-SARS-CoV-2 IgG antibodies was 78.9% (581/736; 95% CI: 75.8–81.7) and that of anti-SARS-CoV-2 IgM antibodies was 10.3% (76/736; 95% CI: 8.3–12.7).

### Potential factors associated with anti-SARS-CoV-2 IgG or IgM antibodies

In rural area anti-SARS-CoV-2 IgG or IgM antibodies were detected in 76.7% (188/245) of the enrolled school-aged children, while in peri-urban area they were detected in 80.0% (192/240) and in urban area, they were detected in 85.3% (214/251). According to the adjusted logistic regression model, area was the only characteristic associated with anti-SARS-CoV-2 IgG or IgM antibodies, and was more likely to be present in school-aged children from the urban area, than in school-aged children from the rural area (adjusted odds ratio: 1.679; 95% CI: 1.060–2.684; p-value = 0.028; Table 2).

**Table 2:** Frequencies and unadjusted and adjusted odds ratios for anti-SARS-CoV-2 IgG or IgM in school-aged children in Maputo

Characteristic	% (n/N)	OR (95% CI)	*p*-value	AOR (95% CI)	*p*-value
**Area**					
Rural	76.7% (188/245)	Reference		Reference	
Peri-urban	80.0% (192/240)	1.213 (0.787 - 1.875)	0.383	1.213 (0.785 - 1.880)	0.385
Urban	85.3% (214/251)	1.754 (1.114 - 2.789)	**0.016**	1.679 (1.060 - 2.684)	**0.028**
**Sex**					
Male	78.9% (276/350)	Reference			
Female	82.4% (318/386)	1.254 (0.869 - 1.811)	0.227		
**Age in years**		0.978 (0.881 - 1.088)	0.677		
Positive for anti-SARS-CoV-2 IgG or IgM; Median (Q1 - Q3) (Min - Max)	8 (7 - 10) (5 - 15)				
Negative for anti-SARS-CoV-2 IgG or IgM; Median (Q1 - Q3) (Min - Max)	8 (7 - 10) (6 - 15)				
**Number of household members**		0.923 (0.848 - 1.006)	0.065	0.919 (0.843 - 1.004)	0.058
Positive for anti-SARS-CoV-2 IgG or IgM; Median (Q1 - Q3) (Min - Max)	5 (4 - 7) (2 - 15)				
Negative for anti-SARS-CoV-2 IgG or IgM; Median (Q1 - Q3) (Min - Max)	6 (4 - 7) (2 - 14)				
**Does the child cover’s mouth and nose with a bent elbow when coughing/sneezing?**					
Yes	82.5% (410/497)	Reference		Reference	
No	77.0% (184/239)	0.710 (0.487 - 1.042)	0.077	0.745 (0.507 - 1.102)	0.137
**Does the child sleeps 1.5 m away from other beds?**					
Yes	82.4% (211/256)	Reference			
No	79.8% (383/480)	0.842 (0.565 - 1.239)	0.389		
**Does the child use public transport to school?**					
Yes	84.6% (148/175)	Reference			
No	79.5% (446/561)	0.708 (0.440 - 1.104)	0.139		
**Did the child had contact with a SARS-CoV-2 case at home?**					
Yes	82.0% (91/111)	Reference			
No/Doesn’t know	80.5% (503/625)	0.906 (0.525 - 1.501)	0.712		
**Did the child had contact with a SARS-CoV-2 case at school?**					
Yes	83.0% (39/47)	Reference			
No/Doesn’t know	80.6% (555/689)	0.850 (0.361 - 1.769)	0.684		
**Did the child had a SARS-CoV-2 positive diagnosis before enrollment in the study?**					
Yes	100.0% (5/5)				
No	80.6% (589/731)	-	0.983		

In school-aged children that covered their mouth and nose with a bent elbow (in a “V” shape) when coughing/sneezing, the prevalence of anti-SARS-CoV-2 IgG or IgM was 82.5% (410/497), and in school-aged children who did not covered their mouth and nose with a bent elbow when coughing/sneezing, the prevalence of anti-SARS-CoV-2 IgG or IgM antibodies was 77.0% (184/239, Table 2).

Among the school-aged children who had a SARS-CoV-2 positive diagnosis before enrollment in the study, 100% (5/5) had a positive anti-SARS-CoV-2 IgG or IgM antibodies, whereas among school-aged children who did not have a SARS-CoV-2 positive diagnosis before enrollment in the study, 80.6% (589/731) had positive anti-SARS-CoV-2 IgG or IgM antibodies.

## Discussion

We conducted a cross-sectional survey between August and November 2022 to assess the prevalence of SARS-CoV-2 antigens and the seroprevalence of anti-SARS-CoV-2 IgG or IgM antibodies in selected schools in Maputo City and Province, Mozambique. The observed seroprevalence was significantly greater than that reported for the same geographic area between July and August 2020, involving similar participants [7]. In Manhiça district, a rural area located approximately 65 km from our study sites, a community survey with three cross-sectional periods reported, increasing seroprevalence of SARS-CoV-2 rates in participants younger than 20 years of age: 25.6% (April-June 2021), 58.2% (October-November 2021), and 82.6% (January-February 2022) [13]. The increase in the seroprevalence of anti-SARS-CoV-2 over time in children may be explained by the number of waves recorded by the country [9–11, 24]. Mozambique reported, four waves between 2020 and 2022, which could increase children’s exposure over time as the number of infected young people increased within the waves [12]. Global prevalence data indicate that SARS-CoV-2 infection in children and adolescents has increased due to the increased circulation of Delta and Omicron variants and increased vaccination coverage against COVID-19 among adults [25]. This high seroprevalence of SARS-CoV-2 indicates that many children were exposed to the virus and developed antibodies that can contribute to the herd immunity and reduce the transmission of the virus in the community and school and reducing the probability of occurrence of outbreaks that can overload the health system [26, 27].

Geographical heterogeneity in the seroprevalence rate was observed, being least common in the school at the rural area compared to the selected schools at the urban area. Increased seroprevalence in urban areas has been noted in other settings compared to that in rural areas, supported by the fact that the antibody rate can decline over time, mostly in unvaccinated populations, in turn, urban areas can present higher population density along with mobility, which may facilitate virus spread in the community [28–30].

The self-reported proportion of SARS-CoV-2 positive cases before study enrollment was similar to the prevalence of SARS-CoV-2 antigens observed in the study (i.e., < 1%). The Manhiça district reported a SARS-CoV-2 incidence ranging from 0.7 to 10.1% between April and June 2021, October to November 2021, and January to February 2022 [13]. Prevalence variation can be explained by the influence of waves on the number of exposed and infected individuals as well as the circulation of variants of concern identified in the country [12]. We observed prior reports of SARS-CoV-2 infection in less than 1% of our sample. The lower proportion of self-reported SARS-CoV-2 positive cases suggests no overcapacity of health facilities due to SARS-CoV-2 in school-aged children and minimal morbidity and mortality.

The self-reported proportion of SARS-CoV-2 positive cases before study enrollment described mild symptoms, most of whom did not require hospitalization. A previous study showed that shortness of breath, fever, headache, cough, and nasal congestion were among the most frequently reported symptoms in children [31]. However, clinical symptoms are less frequently reported in children than in adults [32]. Potential drivers of higher morbidity in adults than in children include differences in immune responses, as adults are more prone to comorbidities as a consequence of alcohol and tobacco abuse as well as obesity [33].

In our results, compliance with non-pharmacological interventions, respiratory hygiene measurements like covering the mouth and nose with a bent elbow and other factors was not associated with seroprevalence status (e.g., usage of public transport to attend in-person classes). Knowledge and compliance with non-pharmacological interventions can impact in the SARS-CoV-2 seroprevalence, however if a person doesn’t wash their bent elbow after coughing/sneezing, it can accumulate the viral particles and consequently increase the virus transmission risk, therefore limiting its effectiveness [34].

Non-pharmacological interventions were set up after the first COVID-19 case was diagnosed in the country, with the aim of limiting virus transmission, slowing community transmission, preventing health care system saturation and later collapse. Available data indicate that the positive effect of non-pharmacological interventions is associated with a decrease in the death rate and not with the spread of the virus [2, 35, 36]. Notably, our study was implemented after four waves were reported in the country, which increased the seroprevalence rate, over time, as similarly reported elsewhere [9–11, 24, 25].

A single school was expected for each area (rural, peri-urban, and urban). However, the minimal sample size was not achieved in the first selected urban school. Thus, a backup school (located in the same area) was added to ensure that the minimum sample size was obtained from each area. To minimize bias, a sensitivity analysis to assess seroprevalence and prevalence differences between the two schools in the urban area was conducted before analyzing the two schools as an area. Several biases, in particular, selection bias and social desirability bias, may occur in this type of study, limiting the generalizability of the findings to Maputo City and Province, as well as each of the studied areas (rural, peri-urban, and urban).

None of the enrolled schools had a surveillance or monitoring system that would enable tracking and registration of suspected/confirmed SARS-CoV-2 cases. With the reopening of schools, a monitoring system would be helpful for detecting cases early and mitigating the potential spread of the virus among school-aged children. In addition, it is essential to assess the health and sanitation situation in schools and promote good hygiene practices that promote healthier conditions, not only against SARS-CoV-2 but also against other preventable infectious diseases.

## Conclusions

One year after the in-class teaching period resumed, active SARS-CoV-2 cases in school-aged children were observed in the peri-urban and urban areas of Maputo City, Mozambique. More than half of the school-aged children were exposed to SARS-CoV-2, with significantly higher prevalence in urban schools compared to rural schools in Maputo City and Province. Less than 1% had a SARS-CoV-2 positive diagnosis before enrollment in this study, suggesting that over time primary school-aged children may be exposed to the virus and that symptomatic cases have a minimal incidence.

### Electronic supplementary material

Below is the link to the electronic supplementary material.

Supplementary Material 1

Supplementary Material 2

Supplementary Material 3

## Data Availability

The data used for this analysis can be obtained from the Instituto Nacional de Saúde, Mozambique, through the corresponding author. Researchers interested in secondary analysis must submit a research proposal for consideration by the study investigators, as well as by the Directorate for Research in Health and Well-Being. Upon approval, the requestor must sign a data use agreement.

## Abbreviations

AOR: Adjusted Odds Ratio
CI: Confidence Interval
COVID-19: Coronavirus Disease 2019
IgG: Immunoglobulin G
IgM: Immunoglobulin M
OR: Odds Ratio
SARS-CoV-2: Severe Acute Respiratory Syndrome Coronavirus 2
