# Supplementary material for: SARS-CoV-2 in Mozambican primary school-aged children at Maputo City and Province: a cross-sectional study from a low-income country
Source: BMC Pediatr. 2024 Jul 2;24:425. doi: 10.1186/s12887-024-04904-x (PMC11221092; doi:10.1186/s12887-024-04904-x)
Supplement: Supplementary file 1 — Supplementary Material 1 [file 12887_2024_4904_MOESM1_ESM.docx]

**Case Report Form**

| Participant code: __________________ |  |
| --- | --- |
| Type of area: Rural \|__\| Peri-urban \|__\| Urban \|__\|  School name: _____________________________________________________________ | |
| Classroom number: \|___\| | Grade: \|__\| |
|  |  |
| 1. **Sociodemographic information** | |
| - 1. Date of birth (Day/Month/Year): __/___/____ |  |
| - 1. Inclusion date (Day/Month/Year): ___/___/____ |  |
| - 1. Age (in years): ______ (If date of birth is unknown) | |
| - 1. Child sex: Male \|__\| Female\|___\| |  |
| - 1. How many persons live in the child’s house (*including the child*)? | |
| - 1. Mid-Upper-Arm Circumference in centimeters (*measured by the study personnel*): \|____\| | |
| 1. **Participant symptoms** |  |
| 2.1. Body temperature (ºC) (*measured with infra-red thermometer*) \|___\| | |
| 2.2. Does the child have any discomfort, such as: headache, pain in the body, nasal congestion, fatigue, loss of taste, loss of taste, or other? Yes \|__\| No \|___\| | |
| 2.3. If **Yes on 2.2**, list the symptoms: _____________________________________________________  ____________________________________________________________________________________ | |
| 1. **Practices and attitudes related to SARS-CoV-2** | |
| 3.1. Ask the child to demonstrate how she proceeds when coughing or sneezing. (*Observe if the child cover’s the mouth and nose with a bent elbow*):  Knows how to proceed when coughing/sneezing \|__\|  Does not know how to proceed when coughing/sneezing \|___\| | |
| 3.2. Does the child sleeps 1.5m away from other beds *(Ask if the child sleeps with another person in the room, if yes, ask to indicate the distance between where the child sleeps compared to the roommate)*. Yes \|__\| No \|___\| | |
| 3.3. Did the child had contact with a SARS-CoV-2 case at home? Yes \|__\| No \|___\| | |
| 3.4. Did the child had contact with a SARS-CoV-2 case at school? Yes \|__\| No \|___\| Doesn’t know \|__\| | |
| 3.5. Does the child use public transport to attend school classes? Yes \|__\| No \|___\| Doesn’t know \|__\| | |
| 3.6. Did the child had a SARS-CoV-2 positive diagnosis before enrollment in the study? Yes \|__\| No \|__\| | |
| 3.6.a. If yes on 3.6. indicate the date (Day/Month/Year): __/___/___ | |
| 3.6.b. If yes on 3.6. did the child had symptoms? Yes \|__\| No \|___\| | |
| 3.6.c. If yes on 3.6.b. describe the symptoms the child had: ___________________________________  ____________________________________________________________________________________ | |
| 3.6.d. If yes on 3.6. was the child hospitalized due to COVID-19? Yes \|__\| No \|___\| | |
| 1. **Point of-care testing at school** | |
| - 1. Anti-SARS-CoV-2 IgG antibodies result: Negative \|__\| Positive \|__\| Not performed \|__\| | |
| - 1. Anti-SARS-CoV-2 IgM antibodies result: Negative \|__\| Positive \|__\| Not performed\|__\| | |
| - - 1. If anti-SARS-CoV-2 antibodies test not performed, describe the reason: _____________________________________________________________________________   ____________________________________________________________________________________ | |
| - 1. SARS-CoV-2 antigen result: Negative \|__\| Positive \|__\| Not performed \|__\| | |
| - - 1. If SARS-CoV-2 antigen test not performed, describe the reason: _________________________________________________________________________   ________________________________________________________________________________ | |
| 1. Comments: __________________________________________________________________   ________________________________________________________________________________  ________________________________________________________________________________ | |
| Case report form filled by:_________________________________Date (Day/Month/Year): __/__/____ | |
| Case report form validated by:______________________________Date (Day/Month/Year): __/__/____ | |
